# Usefulness of SPINA model in evaluation of the thyroid function in euthyroid pediatric patients children with subclinical hypothyroidism

**DOI:** 10.3389/fendo.2025.1365354

**Published:** 2025-03-31

**Authors:** Aristeidis Giannakopoulos, Alexandra Efthymiadou, Dimitra Kritikou, Dionisios Chrysis

**Affiliations:** Division of Pediatric Endocrinology, Department of Pediatrics, Medical School of Patras, University Hospital, Patras, Greece

**Keywords:** pediatric endocrinology, thyroid, subclinical hypothyroidism, multivariate models, TSH

## Abstract

**Objective:**

Subclinical hypothyroidism (SH) is biochemically defined by increased TSH and normal thyroid hormones, and its management is a matter of debate. Herein, we investigated thyroid function in euthyroid and children with SH using a structure parameter inference approach (SPINA) model along with published data from population-based TSH-FT4 curves.

**Design:**

The study included 183 children and adolescents with SH and 313 healthy controls. The predicted and actual secretory capacity of thyroid gland (SPINA-GT) was calculated in all euthyroid children divided into quartiles according to TSH values, and in children with SH, which were further subcategorized into those with mild SH (TSH: 4.5 – 10 mIU/L) and severe SH (TSH > 10 mIU/L).

**Results:**

Actual SPINA-GT values decreased significantly from the 1^st^ to the 2^nd^ quartile of normal TSH values in euthyroid children (p< 0.001). It was also significantly decreased in mild SH compared to the upper 2 TSH quartiles of euthyroid range, and in severe SH compared to mild SH. Actual SPINA-GT values were significantly decreased from predicted SPINA-GT values in the upper 2 quartiles of TSH in euthyroid children and in children with mild and severe SH. Thyroid antibody positivity was statistically higher in the SH group (11.3%) compared to the euthyroid group (6.4%).

**Conclusion:**

The implementation of SPINA model for thyroid function gives a wider perspective of thyroid gland’s performance within the euthyroid range of TSH, as well as in SH and add to the discussion for the nature of SH, and the necessity of its management.

## Introduction

In current pediatric clinical practice, the diagnosis and management of typical thyroidopathies such as overt hypo- or hyperthyroidism is an unambiguous task, thanks to the presence of sensitive assays for serum TSH and FT4 concentrations. However, a state of a mild thyroid derangement that is often encountered in pediatrics, called subclinical hypothyroidism (SH), is defined by serum TSH level above the upper limit of the reference range (TSH >4.5 mIU/L) in the context of normal serum concentrations of total thyroxine (T4) or free T4 (FT4) (1, 2). After the neonatal period, SH is further subcategorized by serum TSH levels, in a mild (TSH 4.5 to 10 mIU/L) or a severe form (TSH > 10 mIU/L) (1). While progression to overt hypothyroidism is possible, more commonly, stable persistence or even spontaneous normalization of the elevated TSH is observed during follow up (3). Diagnosis of SH is usually not based on specific clinical signs or symptoms but rather emerges as an incidental finding in a laboratory workup. Furthermore, the management of SH that may include either initiation of treatment with thyroxine or simple monitoring of the thyroid function, is still a matter of debate (4).

The normal range of serum TSH levels in children and adolescents is well defined and displays a skewed distribution (5). Serum concentrations of TSH and T4 (or FT4) are tightly regulated by the thyroid homeostatic mechanisms in a way that minor changes in circulating FT4 concentration result in large relative changes of TSH (6). Several studies had initially described the TSH - FT4 relationship as an inverse log linear one (7–9) although later population-based studies described the TSH - FT4 correlation by 2 overlapping negative sigmoid curves with some variations attributed to age and sex differences (10). The study of hypothalamo-pituitary –thyroid physiology over the years demonstrated the existence of the so-called set-point of thyroid function, which reflects the individual characteristics of both thyroid gland response to TSH stimulation, and feedback loop of thyroid hormones to hypothalamus and pituitary. This was based on evidence showing that individual reference ranges for serum T3 and T4 displayed half the width of population-based reference ranges (11). Consequently, the intraindividual variability of TSH and FT4 was much narrower than the interindividual one. Later studies supported that thyroid set points are in some degree genetically determined (12–14) and aging may lead to their transposition (15, 16). Recent work by Fitzgerald et al. shows that feedback regulation of TSH and FT4 is much more complex than inferred by the published FT4 and TSH curves and provides evidence that pituitary sensitivity to FT4 and thyroid sensitivity to TSH are linked, resulting in individualized response combination curves (17).

Based on the critical effect of thyroid hormones on brain development and metabolic homeostasis, the yet unanswered questions of SH in childhood regards the probability of any long-term adverse effects on central nervus system development mainly during infancy (18–20), or on the healthiness of cardiovascular system at older ages (21–24). The contradictory results of studies that have addressed these questions sustain the uncertainties on this matter.

To investigate more in depth the nature of SH in children, we studied the thyroid function of both euthyroid and children with SH by implementing a previously published multiparametric model which follows a structure parameter inference approach (SPINA) that includes the pituitary – thyroid feedback control (25). Our aim is to consider the hidden physiological parameters that govern the thyroid function in the euthyroid range of TSH values as well as in SH. Data from a large population-based analytical model that describe the TSH - FT4 relationship in both euthyroid and children with SH was used as reference for the results of our population study (10, 26). More specifically, we calculated the TSH values (henceforth referred to as predicted TSH) of all subjects using the equations developed by population -based TSH – FT4 data (10). Then, we calculated the thyroid production capacity using the SPINA model (25, 27). This latter model has evolved step-wisely over the years, from the standard logarithmic model of thyroid homeostasis (28), to more detailed multi-dimensional and non-linear relationships between TSH, FT4, T3 and their binding proteins (27, 29, 30). Descriptively, this platform includes the Michaelis–Menten kinetics for the deiodination of T4 with a time-delay model (thyroid), a negative exponential model for feedback inhibition of TSH release, and a non-linear description of plasma protein binding (31, 32). The thyroid’s secretory capacity (SPINA-GT), also referred to as thyroid output or thyroid capacity, provides an estimate for the maximum secretion rate of thyroid hormone under stimulated conditions (25). The development of SPINA-GT model was promoted by a long-standing reflection on the non-improvement of hypothyroid symptoms in a fraction of patients that were under treatment with levothyroxine and, nonetheless, biochemically euthyroid (33, 34). The SPINA parameters have been validated in several studies in different populations with more than 10,000 subjects (35, 36). To the best of our knowledge, no studies have been published regarding SPINA implementation for thyroid function in children. Finally, we tried to interpret the results of classical thyroid analysis under the prism of multivariate modeling.

## Methods

This is a retrospective study that included 183 children and adolescents of Greek origin with SH with age 7.25 years (median) (range: 1 – 14.9 years) and 313 healthy children with age 8.5 years (median) (range: 1.3 – 16.7 years). SH was defined as serum TSH > 4.5 µIU/L (min = 4.51 and max = 15) with FT4 levels within normal range (min = 10.43 pmol/L and max = 24.5 pmol/L). The euthyroid group consisted of children and adolescents who presented to our department due to parental concern for short stature or for thyroid evaluation due to positive family history for thyroid disease. Subjects with known chronic disease, specific or non-specific clinical symptomatology or receiving any medication were excluded from the study. The study was approved by the local Ethical Committee of the University Hospital of Patras, Greece. Written informed consent for participation in this study was provided by the patient’s’ parents. All our subjects were considered as iodine sufficient, since Greece belongs to the group of countries with adequate iodine nutrition (37). Date of birth, family history of thyroid disorders and any acute or chronic disease were recorded. Body, height, and weight were measured using standard anthropometric techniques. BMI was calculated using the formula weight/height ^ 2 (kg/m^2^). BMI standard deviations score (SDS) was computed for each subject using the formula: BMI-SDS = (actual BMI-mean BMI for age, race and gender)/BMI SD for age and gender.

Serum TSH, FT4, Thyroglobulin (TG-Ab) and thyroid peroxidase (TPO-Ab) antibodies were measured by electro-chemiluminescence (Elecsys 2010, Roche Diagnostics). Anti-thyroid antibody status was considered positive when TG-Ab and/or TPO-Ab was positive (>34 IU/mL).

The relationship between the natural logarithm of TSH and free T4 in subjects > 1 year old, not receiving thyroxine treatment, were described by the following equations (10):


(1) $$ln\;TSH\;=\;1.4+\frac{3.5}{1+e^{-(7.0-FT4)/1.0}}\text{ if FT}4<12\text{ pmol}/L$$

(2) $$ln\;TSH=-3.7+\frac{5.3}{1+e^{-(20.6-\text{FT}4/3.0)}}\text{ if FT}4>12\text{ pmol}/\text{L}$$

Predicted *lnTSH* was calculated using the above formulas, and results were back log-transformed to predicted TSH.

The thyroid’s secretory capacity (SPINA-GT) was defined by the formula:


(3) $$G_{T}=\frac{\beta_{T}\;\left(D_{T}\;+\;\left[TSH\right]\right)\left(1\;+\;K_{41}\left[TBG\right]\;+\;K_{42}\left[TBPA\right]\right)\left[FT_{4}\right]}{\;a_{T}\left[TSH\right]}$$

(α_T_: Dilution factor for thyroxine = 0.1 L^-1^ β_T_: Clearance exponent for T4 = 1.1e-6 s^-1^ D_T_: Damping constant (EC_50_) of TSH set to 2.75 mIU/L according to experimental data previously reported (ref) K_41_: Dissociation constant of T4 at thyroxine-binding globulin = 2e10L/mol K_42_: Dissociation constant of T4 at transthyretin = 2e8 L/mol. The dissociation constants depend on the thyroid secretion rate and provide a distinct TSH for any given FT4 level as a function of thyroid secretory capacity. [TBG]: standard concentration of thyroxine-binding globulin = 300nmol/L [TBPA]: Standard transthyretin concentration = 4.5 μmol/L) as a function of equilibrium concentrations of TSH, free T4, and constants or measured values for dissociation, protein binding, distribution, and elimination (25). The reference range of SPINA-GT is between 1.4 and 8.7 pmol/s (38).

### Statistics

Normally distributed parametric data were presented as mean ± SD and were compared with the two-sided Student’s t-test. Statistical comparison of SPINA-GT among groups defined by different TSH values was performed with analysis of variance (ANOVA) with Tukey’s *post-hoc* test and reported adjusted P values. Statistical analysis of thyroid antibody positivity between groups was performed by Pearson’s chi-squared test. A p value of <0.05 was considered statistically significant in all instances. For the analysis, Stata (version 16) was used.

## Results

All 496 subjects (Male/Female: 229/267) had a median age of 8.17 year (range 1.1 to 16.75 years old). The SH group (n=183) was of younger age than the euthyroid group (n=313) (7.46 vs 8.6 years, p<0.05). BMI SDS was not statistically different between the 2 groups (**Table 1**). Analysis of thyroid function in both groups using serum TSH and FT4 measurements showed higher TSH (p<0.001) and lower FT4 serum levels in SH group (p<0.05) as expected. Thyroid antibody positivity was statistically higher (11.3%) in the SH group (n=22/183) compared to the euthyroid group (6.4%) (n=20/313) (**Table 1**).

**Table 1:** Demographics, BMI, and biochemical indices of thyroid function in children with SH and euthyroid children (Statistical significance denoted with asterisks: **p<0.001 *p<0.05).

	SH group	Euthyroid group
Individuals	183	313
Age (years)	7.46 ± 3.6*	8.6 ± 3.47
Sex (M/F)	84/99	145/168
BMI SDS	1.11 ± 1.46	1.03 ± 1.59
TSH (mIU/L)	6.7 ± 2.05**	3.01 ± 0.95
FT4 (pmol/L)	15.7 ± 2.32*	16.3 ± 2.19
Individuals with TG-Ab (+) AND/OR TPO-Ab (+)	22 (11.3%)*	20 (6.4%)

**Figure 1:**
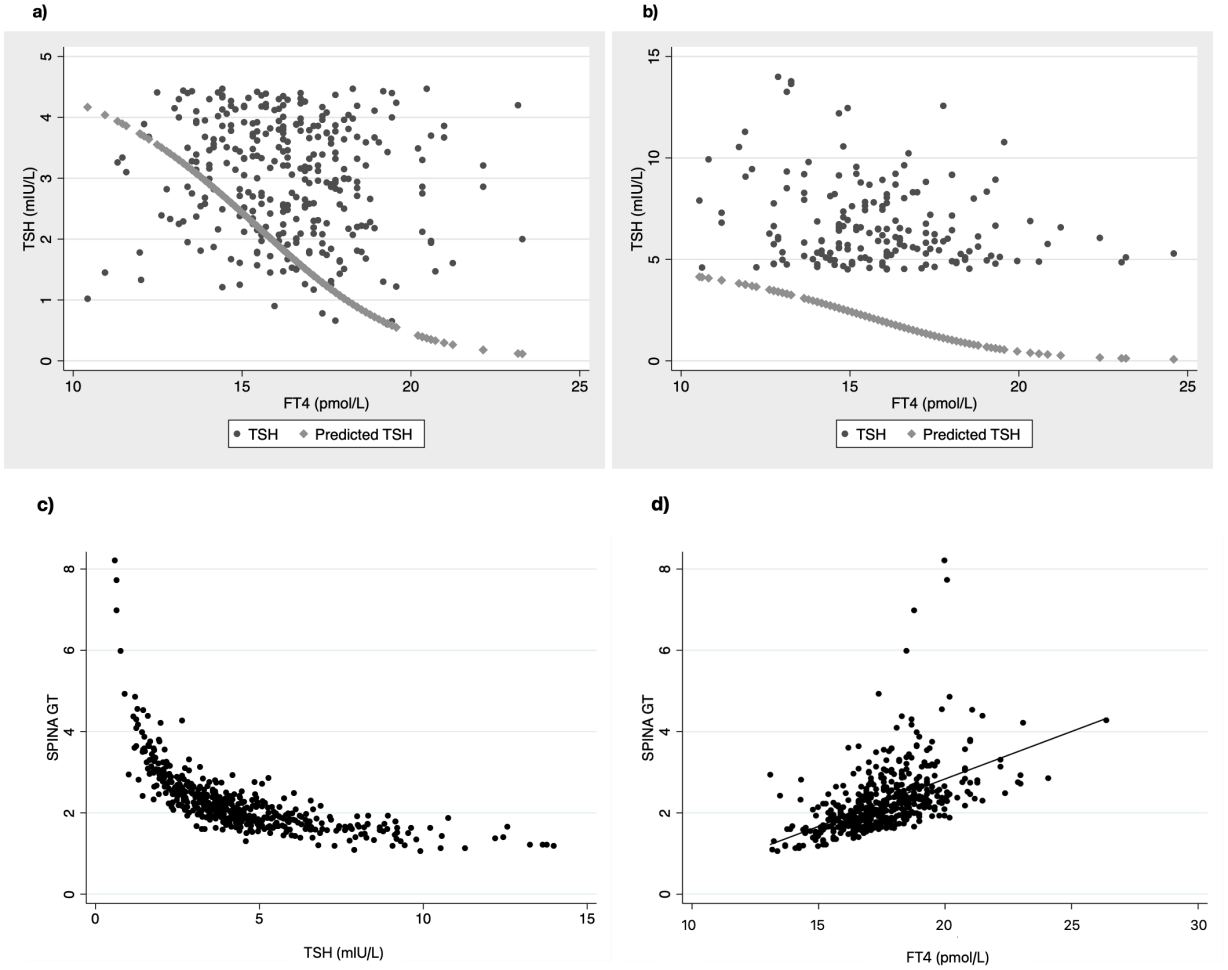
Scatterplots of actual TSH values dispersed around the line of predicted TSH in relation to FT4 values in **(a)** euthyroid children and **(b)** children with SH. Correlation of SPINA-GT to TSH **(c)** and FT4 **(d)** in all children (euthyroid and SH group).

**Figure 2:**
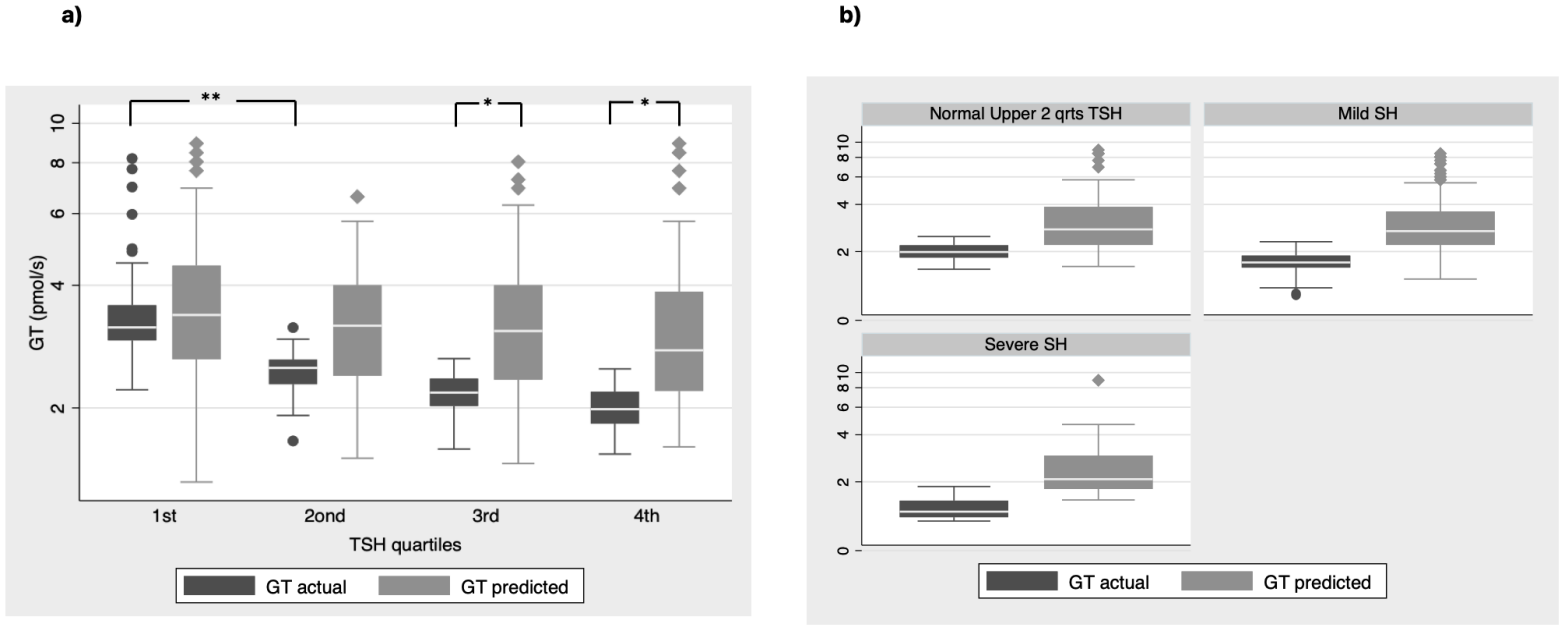
SPINA GT actual vs predicted values **(a)** in all euthyroid children divided in groups by quartiles of TSH values and **(b)** in children with normal TSH in the upper 2 quartiles and children with mild and severe SH (**P < 0.001, *P<0.01).

Firstly, we used the Equations 1, 2 (see methods section) that derived from the population curves of TSH and FT4 relationship (10) to produce the set of predicted TSH values that correspond to the FT4 values of our subjects belonging to either euthyroid or SH group. With this approach we tried to estimate the dispersion of TSH values around the model-predicted ones for the SH group, compared to the euthyroid group. The measured TSH values against the predicted ones were plotted for both euthyroid and SH group in **Figure 1** (a and b respectively). Actual TSH values were dispersed around the curve of predicted TSH values within the entire euthyroid range, whereas the actual TSH values of children with SH were all above the predicted TSH curve. We then implemented the SPINA-GT formula on all children (euthyroid and SH group), using the actual univariate TSH - FT4 paired values for each subject, and the plots of SPINA-GT values in relation to the measured TSH (**Figure 1c**) or FT4 (**Figure 1d**). SPINA-GT values in correlation to the TSH values of the entire range (euthyroid and SH) formed an L- shaped scatterplot that showed a steep decrease within the lower range of TSH values, followed by a gradual decrease of GT towards the higher TSH values (**Figure 1c**). Additionally, SPINA-GT values were significantly correlated to FT4 levels (r=0.523, p<0.001) (**Figure 1d**). Subsequently, we analyzed the SPINA-GT levels in euthyroid children divided into 4 groups defined by TSH quartiles and in children with mild and severe SH. **Table 2** shows the SPINA-GT median and mean values in each TSH quartile delimited by a min and a max TSH value. We found that within the euthyroid range of TSH values, actual SPINA-GT was significantly lower in the 2^nd^ quartile compared to the 1^st^ quartile (p<0.001) while the lower values of actual SPINA-GT seen in the 3^rd^ and 4^th^ TSH quartiles were not statistically different compared to the 2^nd^ quartile (**Figure 2a**). Children with SH showed significantly lower actual SPINA-GT levels compared to the actual SPINA-GT levels of upper 2 TSH quartiles in the euthyroid group (p <0.001). Respectively, the group with severe SH had significantly lower levels than children with mild SH (p<0.001) (**Figure 2b**).

**Table 2:** TSH values (min and max) in euthyroid (by quartiles) and children with SH and their corresponding SPINA-GT value (median and mean) along with the frequency of thyroid antibody positivity.

Euthyroid Quartiles	TSH min (µIU/L)	TSH max (µIU/L)	SPINA-GT median (pmol/s)	SPINA-GT mean (pmol/s)	Individuals with positive thyroid antibodies
1^st^	0.6	2.25	3.21	3.49	3.7% (3/80)
2^nd^	2.26	3.14	2.53	2.52	3.9% (3/77)
3^rd^	3.15	3.82	2.20	2.22	10% (8/78)
4^th^	3.85	4.47	1.70	2.03	7.7% (6/78)
Mild SH	4.5	9.93	1.77	1.74	12% (20/171)
Severe SH	10.23	14	1.29	1.36	17% (2/12)

Next, we compared the previously reported SPINA-GT levels (referred also as actual SPINA-GT levels) to the predicted SPINA-GT levels (calculated from the predicted TSH using the Equations 1, 2 described in methods) in both euthyroid and SH groups. We observed that in euthyroid children, predicted SPINA-GT levels were significant higher only in the 3^rd^ and 4^th^ TSH quartiles (p<0.001). (**Figure 2a**). In the SH group predicted SPINA-GT levels were significantly higher in both mild and severe SH compared to the actual SPINA-GT levels. (**Figure 2b**) Regarding thyroid antibody positivity, individuals with SH show significantly increased positivity in thyroid antibodies compared to the euthyroid TSH range (p<0.05). (**Table 2**)

## Discussion

Subclinical hypothyroidism in children, a biochemically defined condition with no clinical symptomatology, is a puzzling entity regarding its biological significance and its management. The debate on simple monitoring or treatment with levothyroxine in SH originates mainly from data on probable long-term implications on neurodevelopment when diagnosed in very young age, given the effect of thyroid hormones on postnatal brain development (39). The worse neurocognitive outcome found in school-age children, who had TSH values from 99.5th and 99.9th percentiles at neonatal screening in a large population study (40), argues in favor of treating SH in the very young ages. However, this finding is not ubiquitously supported by other smaller studies (20, 41). In cardio-metabolic health, higher lipid levels and more specifically non-HDL C, triglycerides and total cholesterol have been found to be significantly higher in SH by many studies (21, 23, 24, 42). On the contrary, blood pressure (43, 44) and glucose metabolism (21, 42) have not shown any consistent correlation with the increase of TSH in the pediatric population.

The first observation of our study was the large dispersion of TSH values around the fit line of predicted TSH values in euthyroid children. This is in accordance with the concept of increased variability of thyroid set points within the euthyroid state, that is every individual has his own homeostatic relationship between TSH and FT4. The pituitary – thyroid setpoint has strong genetic determinants but its physiology is poorly understood (45, 46). On the contrary, all children with SH had TSH values within the area above the fit line of the predicted TSH (**Figures 1a, b**) which is expected, because SH is defined by TSH values > 4.5 IU/ml.

Next, we investigated the thyroid capacity across the entire euthyroid range of TSH and SH by applying the multiparametric model to our data. For this we used the SPINA-GT model, the *in vivo* evaluation of which has proven in adults, that it can clearly differentiate between the primary thyroid disorders and euthyroidism (30). First, the significant drop of SPINA-GT values between the 1^st^ and 2^nd^ TSH quartile of euthyroid range (**Figure 1c**, **2a**) supports the different thyroid hormone output within the euthyroid range, that is not detected by the classical univariate analysis. This part of thyroid physiology can only be thoroughly evaluated by adding to the secretion dynamics, the sum activity of peripheral deiodinases, the enzymes that regulate the peripheral action of thyroid hormones. For example, a parallel increase of type 2 deiodinase activity at the tissue level could compensate for the lower central thyroid production of FT4. This is only partly reflected by the circulating T3 concentrations, since other cellular factors that regulate T3 transport across cell membrane affect the plasma T3 in relation to the intracellular T3 concentration. The latter effect can be estimated by the SPINA-GD model that includes T3 as a variable (25). This is a limitation of our study since we did not have T3 measurements in our subjects, which may be attenuated because FT4, as a factor that takes part in the homeostasis of both secretion and deiodination, reliably reflects the connection between thyroid production and feedback and has demonstrated a stronger correlation to TSH than T3 (47, 48). Second, significant differences between actual and predicted SPINA-GT in euthyroid range are seen only in the 3rd and 4th TSH quartile. The lower thyroid hormone output capacity (actual SPINA-GT) in comparison to the predicted one (SPINA-GT) within the upper 2 TSH quartiles of euthyroid range could append the conversation of the current TSH upper reference limits in children and probably support a further skewness to the left distribution of TSH values. Such an argument has been previously proposed by a study that supports that TSH reference distribution may be skewed by an occult thyroid dysfunction based on increased thyroid antibodies found in the adolescent group (12-19yr) when they presented with TSH values > 2.5 mIU/L (49). In our study, we found an increased percentage of positive thyroid antibodies in the 3rd and 4th TSH quartile which were similar to the percentage in SH (**Table 2**). This may imply the existence of occult thyroid disease in a small group of individuals even in the euthyroid range of TSH values. In clinical practice, this means that single TSH and FT4 values within the upper normal range, do not necessarily reflect normal thyroid function for a specific individual and thus the usefulness of population-based reference range in the identification of a disease state is limited (7). A repeated, within a few months’ time, TSH – FT4 measurement may help identifying a derangement in thyroid function, given that the expected intraindividual variation of TSH is 50% of the interindividual one (11) and the analytical bias of thyroid hormones is common (50).

Focusing on the SH, our results showed that both in mild and severe SH, the actual SPINA GT values were significantly lower than the predicted ones (**Figure 2b**) supporting the lower thyroid capacity in the SH range. The recent development of thyroid function models has proposed a new level of regulation that include the effect of hormones as trophic factors for their downstream glands. In this context, TSH acts as a growth factor for the thyroid gland and thyroid hormones act as inhibiting factors for the pituitary gland (51). This explains the so-called hysteresis phenomenon in which TSH takes many weeks to normalize after thyroid hormones have returned within the normal range when treatment is started for hypo- or hyperthyroidism (52). Increases in thyroid gland-mass comprise a basic component of a slow adaptation, which may restore the previous thyroid set point value through an increased thyroid hormonal production. The chronic persistence or the gradually increased TSH may indicate either the inadequacy of grand-mass compensation effect or the emergence of an intrathyroidal pathology that with finally render the subclinical state into clinical disease. This hypothesis could raise questions about the role of therapy with thyroxine in the case of SH, since exogenous T4 administration would prevent this kind of reaction by suppressing TSH levels. However, there is evidence of a much higher complexity that governs the thyroid – pituitary feedback that involves linked sensitivities to TSH and FT4 respectively that result to particular combinations of TSH-FT4 curves (17). In a clinical setting though, apart from the requirement for specialized software to perform these calculations, it of prime importance any symptoms referred by the patient that may suggest a thyroid dysfunction, as well as the family history.

A main limitation is that some parameters of the model and especially the dissociation constants have been calculated from TSH-FT4 pair data from adults which would question the validity of extrapolating in the pediatric population. However different constants would be expected to affect more the regrouping of individuals (euthyroid or SH) rather than the intragroup comparisons (SH vs non-SH group). Because this model was implemented for the first time in a pediatric cohort, it is important to mention that the constants TBG and TBPA, used in the model’s equation have the same values in children (53, 54). In each case, data origination and model implementation on pediatric populations are essential for evaluating the above results.

## Conclusions

The implementation of multivariate models such as SPINA-GT for thyroid function gives us a wider perspective of thyroid’s functional status within the euthyroid range of TSH, as well as in the state of SH. In addition, it provides more information for the interpretation of the classical univariate thyroid analysis showing that the maximum secretory capacity of the thyroid gland calculated by SPINA is decreased not only in SH, but even within the euthyroid range of TSH. The above changes in thyroid function need to be confirmed by further studies and introduce further questions regarding their nature in development, their meaning for its long-term effects on health and the role of the potential therapy.

## Data Availability

The raw data supporting the conclusions of this article will be made available by the authors, without undue reservation.
